# Contrast Gain Control in Plaid Pattern Detection

**DOI:** 10.1371/journal.pone.0164171

**Published:** 2016-10-20

**Authors:** Pi-Chun Huang, Chien-Chung Chen

**Affiliations:** 1 Department of Psychology, National Cheng Kung University, Tainan, Taiwan; 2 Department of Psychology, National Taiwan University, Taipei, Taiwan; University College London, UNITED KINGDOM

## Abstract

A plaid is a combination of two gratings whose orientations are orthogonal to each other with the same or similar contrasts. We used plaid patterns as stimuli to investigate the mechanisms underlying the detection of a plaid to understand how the visual system combines information from orientation-selective channels. We used a masking paradigm in which an observer was required to detect a target (either a spiral or a plaid) superimposed on a pedestal. We measured the target threshold versus pedestal contrast (TvC) functions at 7 pedestal contrasts for various target-pedestal combinations with a temporal 2AFC paradigm and a staircase procedure. All TvC functions, except the one with an orthogonal spiral pedestal, showed a dipper shape, although the position of the dip and the slope varied across conditions. The result can be explained by a multiple-mechanism divisive inhibition model, which contains several orientation-selective mechanisms. The response of each mechanism is the excitation of a linear filter divided by a broadband inhibitory input. The threshold is determined by a nonlinear combination of the responses of those mechanisms. Alternative models with mechanism(s) specific for plaid did not provide a better description of the data. Thus, a plaid pattern is mediated by a combination of orientation-selective mechanisms. An early plaid-specific mechanism is not necessary for plaid detection.

## Introduction

Much of our understanding of early visual processing comes from experiments using periodic patterns, such as sine-wave gratings or Gabor patches, as stimuli. The reason for using such stimuli is to maximize the response of neurons in the primary visual cortex whose receptive fields contain elongated excitatory and inhibitory regions [1–3]; such a receptive field resembles a wavelet modulated along a specific orientation [4]. Orientation-selective mechanisms were also found underlying visual detection and discrimination [5–8]. In a natural scene, an image is more complicated than a periodic pattern. A natural image contains numerous objects that are, in turn, composed of numerous image features. Hence, to comprehend human visual processing, it is crucial to understand how the visual system integrates individual image features into a coherent percept of an object. There are several approaches to this issue. One of the simplest approaches is to study how the visual system perceives a plaid [9–12]. A plaid is a combination of two gratings whose orientations are orthogonal to each other with the same or similar contrasts; plaids offer vision scientists a simplified model for studying how the visual system processes a scene containing multiple image components with different orientations.

Plaid patterns have some special properties that make them interesting stimuli for probing the visual system. First, the perceived structure of a plaid is altered with contrast. When the contrast of the components (the gratings) is low, a plaid pattern is perceived to resemble two overlapping gratings, and the perceived orientation is the same as that of its components. On the contrary, when the contrast of its components (gratings) is high, a plaid looks like a checkerboard and the perceived orientation is equal to the average of the component orientations [13]. Second, the contrast response elicited by a plaid cannot simply be explained by a linear combination of the responses produced by its components [9, 14, 15]. The detection threshold for a plaid in terms of component contrast can be predicted with probability summation of the detection thresholds of its component gratings [14, 15]. The perceived contrast of plaid was approximately 3 dB less than the sum of its components at the suprathreshold level [14]. A plaid mask can have a profound effect on the detection of other patterns and such an effect can only be predicted by a nonlinear combination of the effects of both vertical and horizontal grating components [9]. These results suggested that component contrasts are not perceived independently, but may be combined nonlinearly.

The visual performances of a plaid and a grating can be quite different. Georgeson and Meese (1997) showed that adapting to a grating that bisected the angle of a plaid or adapting to a plaid that bisected the angle of a plaid tended to cause the perception of a checkerboard-like plaid to break into its oblique component parts [11]. McGovern and Peirce (2010) showed a plaid adaptor decreased the perceived contrast of a plaid target only when the contrast of the plaid target was high [16]. Conversely, a grating adaptor decreased the perceived contrast of a grating when the contrast of the target grating was low. In addition, unlike grating effects, the plaid adaptation effect was found to be phase-insensitive. Olzak and Laurinen (1999) found that the apparent contrast was phase-sensitive when the center and surround were a sinusoidal grating even when another orthogonal grating (plaid) was added to the surround [17]. However, when the plaid became the target, and the surround was also plaid, the contrast-contrast phenomenon became phase-insensitive. Nam et al. (2009) used a visual search paradigm and found there was a parallel search when a plaid target was searched for among various numbers of component gratings[18]. However, if the spatial frequencies of the components were combined in a way that did not produce a salient plaid structure, the visual search became serial, indicating the existence of a plaid-selective mechanism. Finally, Bruchmann, Hintze, and Vorwerk (2012) used meta- and para-contrast masking paradigms to investigate the time course of feature integration in plaids and found that the visual system took approximately 50 ms to integrate the two components of the plaid [19]. The above results suggest that, at least at the suprathreshold level, gratings and plaids have different visual properties. It seems that, when perceiving a plaid, the visual system treats a plaid as a coherent unit. It is not clear how the visual system achieves this. Several possible mechanisms may be behind this effect. First, it is possible that plaids and gratings are processed by different mechanisms that have different dynamic contrast ranges. A plaid mechanism may be more sensitive than an oriented mechanism at high contrasts while the opposite is true at low contrasts. Second, perception of a plaid may be mediated by a cross-orientation suppression that is not present when the stimulus contains only one orientation. Third, there may be different types of interactions between oriented mechanisms that manifest at different contrast levels. For instance, Chen & Foley (2004) suggested that there are two types of cross-orientation interaction between oriented mechanisms: one is a normalization (the so-called divisive inhibition) pool that receives input from differently-oriented linear filters, and the other is a summation process that integrates responses from oriented mechanisms to create a decision variable[9]. The divisive inhibition is relatively weak at low contrasts [20]. This may allow the effect of the summation process from two oriented mechanisms to be more readily observable. In this case the visual performance would most likely be derived from the summation of two oriented mechanisms. Thus, it is relatively easy to investigate cross-orientation interactions at low contrasts. However, at high contrasts, the divisive inhibition is relatively strong. This has the effect of sharpening the tuning function of individually-oriented mechanisms. As a result, the visual performance may seem to be dominated by plaid-specific mechanisms that form plaid perception.

Because of these contrast-dependent effects in plaid detection, we used a pattern-masking paradigm that estimates the response properties throughout the whole gamut of contrast levels to clarify the mechanisms underlying plaid detection. In our experiments, the detection threshold of a target stimulus was measured in the presence of pedestals of different contrasts. The task of the observer was to determine whether he or she could tell the difference between the pedestal alone (intensity C) and the target plus the pedestal (intensity C+ΔC). A typical result plots the detection threshold of the target (ΔC) against the pedestal intensity (C); this is called the target threshold versus pedestal intensity (TvC) function [6, 9, 21]. When the pedestal and target have the same spatial properties (with the exception of contrast), this function has a characteristic dipper shape; a low contrast pedestal facilitates the detection of the target, and a higher contrast pedestal inhibits the detection of the target. Furthermore, a rising limb with a slope close to 0.6 exists for high-contrast pedestals [6]. The TvC function is commonly explained as the consequence of a contrast transducer, or a contrast gain control mechanism, or both. The TvC function reflects response characteristics in the visual system, and the slope of the response function at C is inversely proportional to the contrast discrimination threshold (ΔC) at base contrast C. Thus, the contrast response function of the visual mechanism can be derived.

It has also been shown that when the orientation of the pedestal is orthogonal to the target, the facilitation disappears, but a relatively weak inhibitory effect is preserved [22]. This effect is termed a cross-orientation suppression. The results also indicate that a contrast gain control process pools the signals from different orientations. Another way to probe the properties of cross-orientation contrast gain control is to measure target discrimination over pedestal stimuli of the same type (orientation) while presenting a fixed contrast mask whose orientation is orthogonal to both the target and the pedestal [22, 23]. We call this experimental paradigm a dual pattern-masking paradigm. This paradigm is intended to lead to an understanding of the properties of suppressive summation in contrast gain control. Such rich information derived from masking paradigms would allow us to clarify the mechanism for combining different types of orientation information, and the mechanisms for detecting plaid.

The aims of our research are (1) to investigate the visual mechanism underlying the detection of a plaid pattern (one possibility being that the detection is mediated by a plaid-specific mechanism and another being that the detection results from a combination of two oriented channels with contrast gain control mechanisms), and (2) to investigate the interactions between oriented channels in contrast gain control mechanisms. Thus, we used single and dual pattern-masking paradigms to compare how different features influence the contrast response function; we adapted Chen and Foley’s multiple-mechanism divisive inhibition model to investigate what mechanisms are suitable to describe our behavioral results [9]. More importantly, we used spiral and spiral-composed plaids, instead of using grating or Gabor, as our stimuli to further investigate the orientation interactions across space and to provide linkage between early- and mid-level visual processing.

## Methods

### Participants

Seven participants (PCH, YSH, NP3, NP4, NP5, NP6, and NP7) with corrected-to-normal vision participated in the experiment. PCH was one of the authors. YSH helped in data collection and knew the purpose of the experiment. The rest of the participants were naïve to the purpose of the experiment. The participants were paid and informed of their rights according to the ethical standards laid down in the Declaration of Helsinki. The consent was approved by the National Cheng Kung University research ethics committee for human behavioral sciences. All of the participants provided their written informed consent to participate in this study.

### Apparatus

Stimuli were presented on a CRT monitor (ViewSonic p95f+) controlled by a VSG 2/5 stimulus generator card (Cambridge Research System Ltd., UK), which provided 15-bit gray-level resolution. The resolution of the monitor was 1024 × 768 pixels with a refresh rate of 120 Hz. The mean luminance was 59 cd/m^2^. The CRT monitor was calibrated with an optical photometer (Color Cal II, Cambridge Research Systems Ltd, UK).

### Stimuli

**Fig 1:**
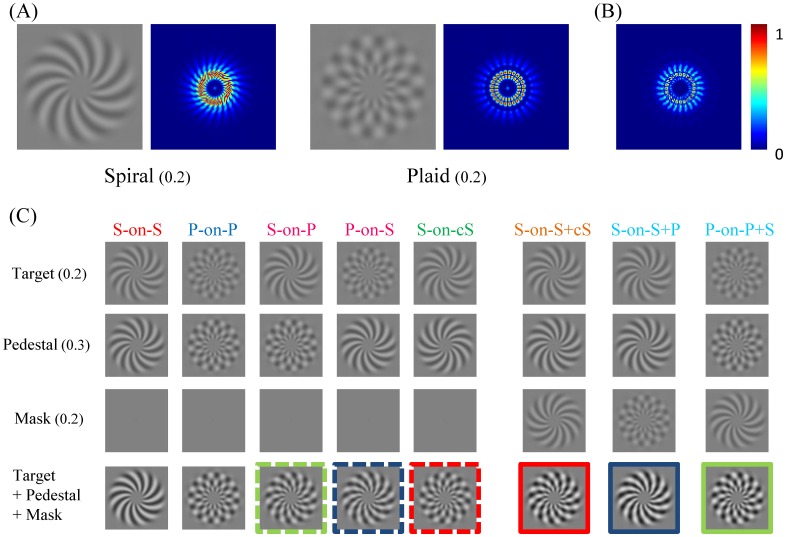
Stimulus demonstration. (A) Stimuli used in the experiment and corresponding amplitude spectra. (B) The difference in amplitude spectra between spiral and plaid shown in (A). (C) Stimulus configurations used in the experiment. S stands for a spiral pattern, P stands for a plaid, and cS stands for a counterclockwise spiral. The number on the left refers to the contrast shown in the figure. The participants reported the difference between (pedestal+mask) and (target+pedestal+mask). To visualize the stimuli for demonstration purposes, the broken colored lines also denote the (pedestal+mask) pairs for the corresponding color configuration of (target+pedestal+mask).

We used spiral and spiral-composed plaids whose orientations were orthogonal to each other. Several reasons motivated the use of spirals. First of all, at any radius, a spiral has the same number of cycles of luminance modulation along its circumference. Thus, the spatial frequency of cycle per degree decreases with the radius. That is, the stimulus is scaled with eccentricity. This property is crucial in initiations such as those that occur in some neuroimaging paradigms, where one must consider cortical magnification factors. Therefore, using spirals as stimuli facilitates association of this study with neuroimaging studies. Second, it is known that neurons in V4 are more sensitive to curved stimuli than to oriented ones [24, 25]. There are also psychophysics studies showing that regarding Glass patterns, a human observer is more sensitive to curved global forms than to oriented ones [26, 27]. Hence, it is more likely for us to be able to tag the properties of mid-level visual mechanisms using spirals. Third, all orientation information is included in both spirals and spiral-composed plaids (see Fig 1(A)). Furthermore, the difference in amplitude spectrum between clockwise spiral and spiral-composed plaid (Fig 1 (B)) is not the amplitude spectrum of the counter-clockwise spiral. Thus, any different behavioral performance that may be found would not be explained by visual processing based on the power spectrum of the stimuli.

The stimuli spanned the range from 1 to 7.7 degrees of eccentricity. The inner and outer edges of the ring were blurred by applying a Gaussian function (SD = 0.72 degrees) to ramp the contrast from its maximum to near zero. Eight stimulus configurations were used in this study. Examples of each configuration are shown in Fig 1(C) and are described as follows:

1. Spiral-on-Spiral (S-on-S): Both the target and the pedestal were spiral patterns with the same spatial frequency, orientation, and phase, but with different contrasts.
2. Plaid-on-Plaid (P-on-P): The target and the pedestal were plaids that were composed of two spiral patterns whose orientations were orthogonal to each other.
3. Spiral-on-Plaid (S-on-P): The target was a spiral pattern, and the pedestal was a plaid.
4. Plaid-on-Spiral (P-on-S): The target was a plaid, and the pedestal was a spiral pattern.
5. Spiral-on-Counter-Spiral (S-on-cS): The target was a spiral pattern, and the pedestal was a spiral pattern with the same spatial frequency, but the orientation was orthogonal to the target.
6. Dual-masking-Spiral-Counter-spiral (S-on-S+cS): The configuration was the same as configuration (1), but a mask was added. The mask was oriented orthogonally to the target and pedestal, and its contrast was fixed at 0.2.
7. Dual-masking-Spiral-Plaid (S-on-S+P): The configuration was the same as configuration (6), but the mask was a plaid and its contrast was fixed at 0.2.
8. Dual-masking-Plaid-Spiral (P-on-P+S): The configuration was the same as configuration (2), but a mask was added. The mask was a spiral, and its contrast was fixed at 0.2.

To achieve independent control of the pedestal and target contrasts, the target and pedestal were interlaced in alternating frames. In the dual pattern-masking conditions, the pedestal and second mask were both present in one frame and the target was present in the other frame. The contrast was measured in Michelson contrast units. For a plaid, the Michelson contrast was equal to the sum of the contrasts of two spiral patterns instead of a single spiral pattern. The contrast of the pedestal was varied (0, 0.0125, 0.025, 0.05, 0.1, 0.2, and 0.4). The highest contrast for the target was 50%. The display was the only light source in the room during the experiment.

### Procedure

A temporal two-alternative forced choice paradigm with a 1-up-3-down staircase procedure was used to measure the detection threshold of the target. The upward and downward step sizes of the staircase were initially set at 25% and 50%, respectively. The downward step size was changed to 12.5% after the first upward reversal. The staircase terminated after 6 upward reversals. The target contrast thresholds for each staircase were calculated by taking the average of the target contrasts at the last five upward and downward reversals.

Observers were cued to the trial onset by an audible tone and by a visual stimulus that was presented for a duration of 100 ms (temporal square pulse). The interstimulus interval (ISI), consisting of a homogeneous mean-luminance field, was 600 ms. A second, similar (cued) stimulus interval followed. Each interval contained a pedestal. A target was randomly presented in one of the two intervals. The task of the observer was to indicate which interval contained the target. After each response, feedback was given by either a high-pitched tone for the wrong response or a low-pitched tone for the correct response. The direction of the spiral (clockwise or counter-clockwise) was randomized for each trail. Seven levels of pedestal contrast were used in the experiment. For each run, one pedestal contrast was randomly chosen for one stimulus configuration. The stimulus configuration was blocked within the pedestal contrasts so the participants was aware the type of the target to look for. We repeated each condition at least three times to derive the detection threshold by averaging the values.

## Results

**Fig 2:**
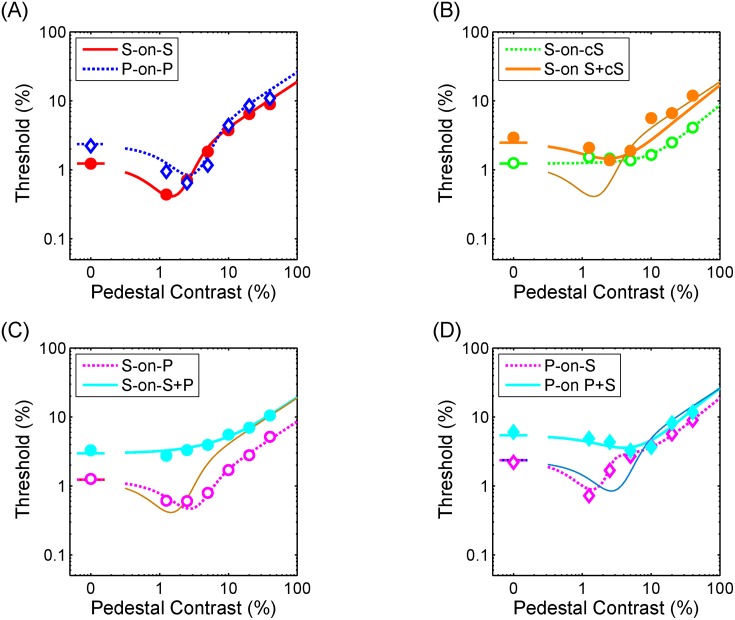
Results for the averaged data whose unit was defined by the pattern contrast. (A) Experimental condition that target and pedestal are of the same stimulus types. (B) Experimental condition that the counter-spiral is used as pedestal or mask. (C) Experimental condition that the spiral is the target. (D) Experimental condition that plaid is the target. See the text for the meanings of each symbols. The smooth curves are fitted curves based on the fit of the model.

Fig 2 shows TvC functions averaged across participants in all tested configurations. The individual data were provided as Supplementary Material (S1 Table). Before averaging, the data for each participant were first multiplied by a scaling constant that made the scaled value of the absolute threshold for the spiral pattern the same as the averaged absolute threshold. The purpose of this scaling was to account for individual differences in contrast sensitivity. Note the scaled factor was only applied to target contrast and not to pedestal contrast. The discrimination threshold of the target as a function of the contrast of the pedestal, the so-called TvC function, was plotted for the averaged data as shown in Fig 2. In Fig 2, the contrast of a spiral and a plaid was defined by their Michelson contrast. The smooth curves are fitted curves based on the fit of the two-mechanism model (see S1A Appendix). Fig 2(A) shows the results for the conditions where the target and pedestal were of the same type. The filled circles represent the thresholds in the S-on-S condition, and the unfilled diamonds represent the thresholds for the P-on-P condition. The TvC function exhibited a typical dipper shape, which is commonly observed in a pattern-masking experiment. That is, the threshold first decreased (facilitation) and then increased (suppression) with the pedestal contrast. The strongest facilitation occurred when the pedestal contrast was at its detection threshold. The paired-t test with Bonferroni correction was performed at each pedestal contrast to test if the discrimination threshold differed. The discrimination threshold was higher for the P-on-P condition at low pedestal contrasts (t (6) = -7.32, p<0.001 for non-pedestal condition and t (6) = -3.47, p = 0.007 for pedestal contrast 0.0125 condition respectively). The P-on-P function converged with the S-on-S function at high pedestal contrast levels(t (6) = -0.63, p = 0.27 for pedestal contrast 0.025, t (6) = -3.36, p = 0.008 for pedestal contrast 0.0125, t (6) = -1.13, p = 0.15 for pedestal contrast 0.1, t (6) = -2.48, p = 0.024 for pedestal contrast 0.2 and t (6) = -0.96, p = 0.19 for pedestal contrast 0.4 condition respectively). The dipper shaped functions were similar to those of the contrast discrimination experiments using oriented Gabor patches in the literature [9, 22]. This suggests an involvement of contrast gain control mechanisms and justifies the use of the spiral and plaid patterns composed of spirals in a pattern-masking study.

Fig 2(B) shows the cross-orientation masking effect. For comparison, the best fit curve for the S-on-S condition shown in Fig 2(A), is replotted here as a thin line. The unfilled circles represent the thresholds in the S-on-cS condition. This TvC function showed no pedestal effect at lower pedestal contrasts. The threshold increased at a high pedestal contrast, but the amount of the threshold elevation was weaker than that of the S-on-S condition(t (6) = -6.15, p<0.001 for pedestal contrast 0.1, t (6) = -8.72, p<0.001 for pedestal contrast 0.2 and t (6) = -9.17, p<0.001 for pedestal contrast 0.4 respectively). The lack of pedestal effect at low contrasts was consistent with the cross-orientation effects reported in previous studies using low spatial frequencies [9, 22, 28] but not in studies with high spatial frequencies [29], even though we used broad band stimuli. The filled circles correspond to the mean thresholds for the S-on-S+cS configuration. This TvC function exhibited a dipper shape at a low pedestal contrast. The S-on-S+cS curve converged with the S-on-S curve at a high pedestal contrast (t (6) = -0.13, p = 0.45 for pedestal contrast 0.05, t (6) = -1.77, p = 0.06 for pedestal contrast 0.1, t (6) = -0.09, p = 0.47 for pedestal contrast 0.2, and t (6) = -2.01, p = 0.05 for pedestal contrast 0.4 respectively). This result is consistent with Foley’s two-mask result [22].

Fig 2(C) shows the TvC function for the spiral target. The unfilled circles represent the thresholds for the S-on-P condition, and the filled circles represent the thresholds for the S-on-S+P condition. The TvC function for the S-on-P condition exhibited a dipper shape and resembled a rightward shifted version of the S-on-S function. This result is consistent with that of Chen and Foley [9], who showed a similar rightward shift for detection of vertical Gabor targets on vertical and plaid pedestals. The TvC function for the S-on-S+P configuration exhibited no dipper shape and no overlap with the S-on-P function. However, it merged with the S-on-S conditions at a high pedestal contrast.

Fig 2(D) shows the results for the plaid target configuration. The unfilled diamonds represent the thresholds for the P-on-S condition, and the filled diamonds represent the thresholds for the P-on-P+S condition. For comparison, the best fit curve for the P-on-P condition, shown in Fig 2(A), is replotted here. The P-on-P+S condition exhibited a shallow dipper shape at a low pedestal contrast and converged with the curve for the P-on-S condition at a high pedestal contrast (t (6) = -2.47, p = 0.025 for pedestal contrast 0.05, t (6) = -0.82, p = 0.28 for pedestal contrast 0.1 and t (6) = -2.62, p = 0.02 for pedestal contrast 0.4). These three TvC functions all converged at a high pedestal contrast.

## Discussion

We used a pattern-masking paradigm to measure TvC functions under different configurations to investigate the visual mechanism underlying the detection of a plaid pattern and the interactions between oriented channels. When the pedestal and target were of the same type (S-on-S and P-on-P), typical dipper functions were found. When the pedestal and target had orthogonal orientations (S-on-cS), typical nondipper shapes were revealed. When the target and the pedestal were of different types, S-on-P and P-on-S, dipper functions were shown, but with different masking strengths than those of S-on-S and P-on-P configurations respectively. Under this dual pattern-masking paradigm, the three TvC functions (S-on-S+cS, S-on-S+P, and P-on-P+S) all converged at high pedestal contrasts.

### Divisive inhibition model for pattern vision

Over the past two decades, the contrast normalization model, or divisive inhibition model [9, 22, 30–33], has been very successful in explaining numerous aspects of human pattern vision. While it is possible to explain the target threshold reduction by the presentation of a low contrast pedestal in terms of uncertainty reduction [34], or threshold elevation in terms of noise increment [35], these models can explain only part of the pattern vision phenomena. We therefore used the divisive inhibition model as a framework to discuss issues regarding plaid perception. There are numerous varieties of divisive inhibition models. The general characteristic of a divisive inhibition model is that the response of a mechanism can be described as the excitation of a linear filter divided by an additive constant plus an inhibitory input, which is a nonlinear sum of the excitations of all relevant filters. The whole model may contain several mechanisms, each of which has a linear filter followed by a nonlinear divisive inhibition process. The performance of an observer is then determined by a nonlinear summation over the responses of relevant mechanisms.

Specifically, we assumed that a set of linear filters operates on stimuli. The excitation of a filter depends on the spatial sensitivity of its profile and the luminance modulation in the stimulus. In our experiment, contrast is a global parameter that does not change with location; thus, the output of the *j*-th linear filter to the *k*-th stimulus pattern can be written as:

$$E\prime_{jk}=Se_{jk}\times c_{k}$$ (1)

where *c*_*k*_ is the contrast of the *k*-th stimulus, and *Se*_*jk*_ is the excitatory sensitivity of the *j*-th filter to *k*-th stimulus.

The excitation of the linear filter is first half-wave rectified [36]:

$$E_{jk}=\text{max}(E_{jk}^{\prime },0)$$ (2)

**Fig 3:**
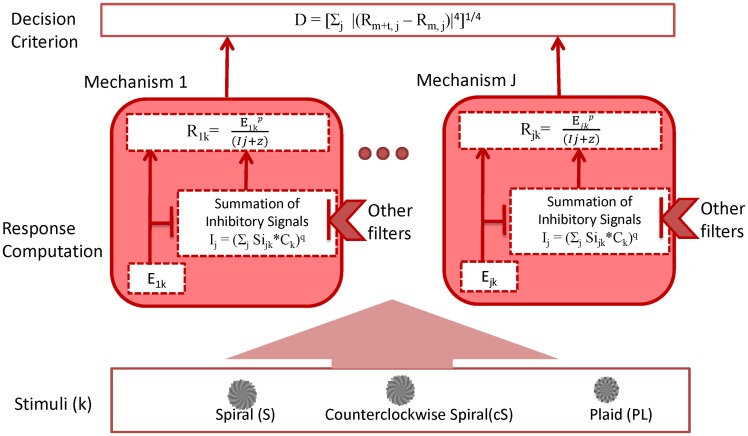
Schematic of the model. See the text for the details.

The inhibition term is a nonlinear combination of the excitations from relevant linear filters [22, 36]. For the *j*-th mechanism, the total divisive inhibition received can be simplified and written as:

$$I_{j}={\left(\Sigma_{j}{\left(Si_{jk}\times c_{k}\right)}^{q\prime }\right)}^{q}$$ (3)

where *Si*_*jk*_ is the inhibitory sensitivity from the stimulus *k* to mechanism *j*, and *q* and *q’* are exponent parameters. We used two exponent parameters to accommodate different summation rules, such as Foley’s Model 2 and Model 3 [22], that are discussed below. The excitation of the linear filter can be nonlinearly transformed to produce the response, *R*_*j*,*k*_, given as:

$$R_{j,k}=\frac{E_{jk}^{p}}{I_{j}+z}$$ (4)

where *p* is an exponent parameter. The denominator contains the divisively inhibitory term *I*_*j*_ and an additive constant *z*. The threshold was determined by the fourth-root summation rule over various mechanisms, given as:

$$d={\left[\underset{j}{\sum }{\left(R_{j,m+t}-R_{j,m}\right)}^{\text{4}}\right]}^{\text{1}/\text{4}}$$ (5)

where *d* comprises the decision variables. The subscript *m+t* denotes the pedestal-plus-target, whereas *m* denotes the pedestal only. If there exists only one mechanism (e.g. spiral), it is Foley’s divisive inhibition model [22]. Fig 3 shows a schematic of the model. The parameters in the model were optimized with a least-square method that minimized the sum of the squared difference between the data and the model predictions in dB units, which is defined as 20 *log(c). We used dB units to compensate the nonlinearity of perceived contrast at different contrast level.

Here, in the context of this general structure, we consider three possible models for plaid pattern perception. In order to explain the main concept of these models clearly, we assumed the contrast response (R) was derived in the absence of pedestal. In other words, the contrast response (R) was equal to the *d’* in Eq 5.

#### A. General mechanism: early summation model

The first model we considered assumed that pattern detection is mediated by a broadly tuned mechanism whose early filter is sensitive to both spiral and plaid. Even if there is a matched filter for each stimulus used in our experiment, this model can be implemented by summing the outputs of various filters before any nonlinearity [11]. This model thus can be called the early summation model.

**Fig 4:**
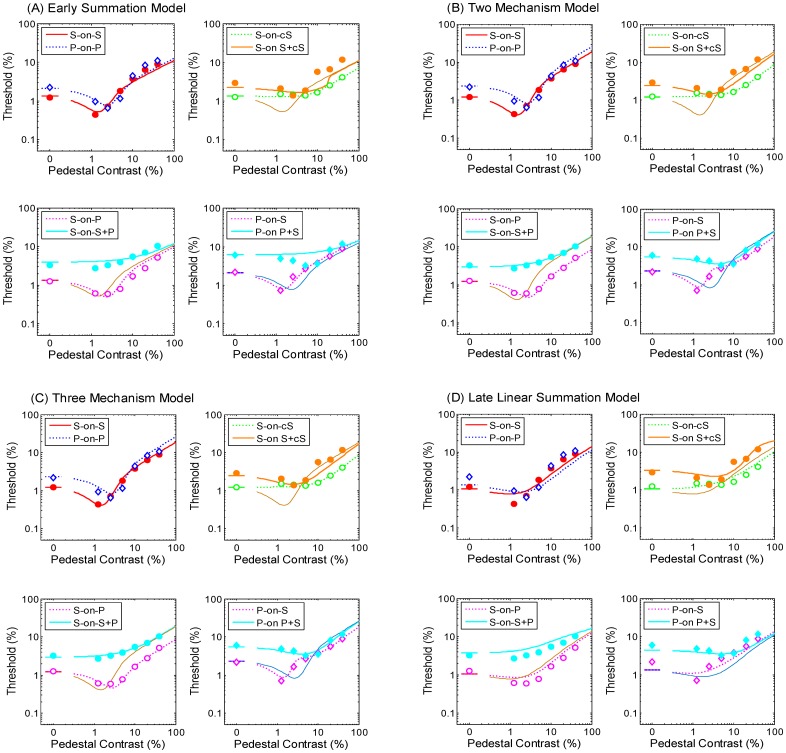
Predictions under four models and the parameters used were derived from the best-fitted parameters. (A) early summation model, (B) two-mechanism model, (C) three-mechanism model, and (D) late linear summation model.

Under this model, there is only one channel in the whole system. Given Eq 4, the response function for the spiral Eq (6) and plaid pattern Eq (7) for this general mechanism can be written, respectively, as follows:

$$R_{S}=\frac{{\left(E_{S}\right)}^{p}}{{\left(I_{S}\right)}^{q}+z}$$ (6)

$$R_{PL}=\frac{E_{PL}{}^{p}}{{\left(I_{PL}\right)}^{q}+z}=\frac{{\left(E_{S}+E_{cS}\right)}^{p}}{{\left(I_{S}+I_{cS}\right)}^{q}+z}$$ (7)

where *R*_*k*_ is the response function for pattern *k* for the general filter. In this model, the same channel responds to both the S and cS patterns. Hence, the effect of a cS pedestal of a particular contrast on S target detection should be the same as the effect of an S pedestal of another contrast. Such equivalent contrasts are determined by the relative sensitivity levels of the channel to these pedestals. Hence, the response functions for S-on-S and S-on-cS conditions should differ in a multiplicative way, thus the corresponding TvC functions should be horizontally shifted copies of each other. Similar arguments also apply to the S and cS components in a plaid. Thus, the S-on-P condition and the P-on-S condition should also be horizontally shifted copies of each other. Fig 4(A) illustrates the predictions of this model. It is obvious that the predictions of this model were not supported by our results. The RMSE of this model was 2.55 dB, which was much larger than the averaged standard error of measurement (0.58 dB).

#### B. Two-mechanism models

Next, we considered the model in which the plaid detection is mediated by two mechanisms with orthogonal orientation tuning such that each has its own contrast gain control processing, as proposed by Chen & Foley [9]. In the context of the current experiment, this model would have two mechanisms whose linear filters are tuned to spiral and counter-spiral patterns respectively. Each mechanism has its own contrast gain control processing, which is calculated by the excitatory response of a linear filter divided by a sum of inhibitory inputs plus a constant. The inhibition contains a self-inhibition component and an inhibitory component coming from the other mechanism. The decision variable is based on the Minkowski summation over the responses of these two mechanisms [37, 38]. The response function of a plaid pattern is:

$$R_{PL}=\sqrt[{1/4}]{{\left[\frac{E_{S}^{p}}{{\left(I_{S}^{}+I_{cS}^{}\right)}^{q}+z}\right]}^{4}+{\left[\frac{E_{cS}^{p}}{{\left(I_{S}^{}+I_{cS}^{}\right)}^{q}+z}\right]}^{4}}=\sqrt[{1/4}]{R_{s}{}^{4}+R_{cs}{}^{4}}$$ (8)

The predictions are shown in Fig 4(B) as well as Fig 2. When the target is spiral, the response function is influenced only by the type of the pedestal. Given the linear summation of the divisive inhibitory terms, the effect of a plaid pedestal (*I*_*S*_ *+ I*_*cS*_) on S target detection is the same as the effect of an S pedestal at a different contrast. Hence, the TvC function for the S-on-P condition should be a horizontally shifted version of the S-on-S TvC function. Similar arguments can be applied to an S-on-cS configuration, but if the divisive inhibition terms (*I*_*cs*_) are influential at low contrast and a fixed decision criterion is applied for detecting a spiral, a non-dipper TvC function is expected. When the target was a plaid, because of the properties of nonlinearity, the responses for that plaid would show the competition between these two mechanisms. Some configurations may show rough curves that may indicate which channel is dominant for those conditions. This model described the performance of the P-on-S configuration especially well where the dipper occurred at a low pedestal contrast. The model predictions are consistent with most of our behavioral results (see S1A Appendix for model implementation) and the RMSE of this model was 1.51 dB, which represent a significant improvement over the general mechanism model.

#### C. Three-mechanism model

Finally, we considered a model in which the detection of spiral and plaid is determined by different mechanisms (three-mechanism model). In addition to the oriented mechanisms for spirals (S and cS), we considered a mechanism tuned specifically to plaids. The plaid mechanism contains a matched linear filter for plaid stimuli and operates in parallel with the other two spiral mechanisms. Thus the response for a plaid can be written as follows:

$$R_{PL}=\frac{E_{PL}^{p}}{{\left(I_{S}^{}+I_{cS}^{}+I_{PL}^{}\right)}^{q}+z}$$ (9)

For a plaid pedestal, the response function was influenced by three divisive inhibition terms (*I*_*S*_, *I*_*cS*_, and *I*_*PL*_). The TvC function for the S-on-P condition should be a horizontally shifted version of the S-on-S TvC function. For a plaid target on a spiral pedestal (P-on-S condition), the divisive inhibition was weaker than that for a plaid pedestal (P-on-P condition) under the same pedestal contrast. Thus one would expect a weaker masking effect for the P-on-S than for the P-on-P condition. The model predictions are shown in Fig 4(C). The RMSE of this model was 1.47 dB, which does not represent a significant improvement over the two-mechanism model (see S1B Appendix for three-mechanism model implementation).

### Is late linear summation possible?

In our two-mechanism model, we used a 4th power Minkowski summation (Quick, 1974) over the responses of the various mechanisms. Meese & Summers [39](2007) proposed a linear summation rule; thus the response function for a plaid could be written as follows:

$$R_{PL}=\frac{E_{S}^{p}+E_{cS}^{p}}{I_{S}^{q}+I_{cS}^{q}+z}=R_{s}+R_{cs}$$ (10)

In the S-on-P condition, as with the S-on-S condition, the decision variable is determined by *R*_*S*_ because without the cS component in the target, *R*_*cS*_ is the same for both intervals and thus is cancelled out when the decision stage computes the response difference between the two intervals. Furthermore, given the linear summation of the divisive inhibitory terms, the effect of a P pedestal on an S target in *R*_*s*_ would be the same as that of an S pedestal at a different contrast. Hence, the TvC function for the S-on-P condition should be a horizontally shifted version of the S-on-S TvC function. The model predictions are shown in Fig 4(D). Some of our experimental results are inconsistent with the model predictions. Quantitatively, compared with our two-mechanism model, the late linear summation model increased the RMSE by more than 71%. Given that the two models have the same number of free parameters, this difference is substantial. Thus, our data does not support the late linear summation model.

### Summation rules for divisive inhibition

In our two-mechanism model, the interactions between the two filter mechanisms occur in two places. The first is a Minkowski summation that integrates responses from oriented mechanisms to create a decision variable Eq (5), as discussed in the previous session. The second is a divisive inhibition signal that is a nonlinear sum of the excitations of linear filters with different orientations Eq (3). There may be different ways to integrate information across mechanisms with various orientations. Foley [22] suggests two ways to compute the divisive inhibition signal. First, the inhibition can be computed as the linear combination of the excitation of each linear filter raised by a power (summation-first rule). This is Model 2 proposed by Foley [22]. This was implemented by fixing *q’* to one in Eq 3. Second, the excitation of each linear filter can be raised by a power before they are added together (power-first rule). That was implemented by fixing *q* to one in Eq 3. This is the same as Model 3 proposed by Foley [22]. Foley found that Model 3 fit the cross-orientation masking data better than Model 2 did [22]. However, Holmes and Meese [23] showed an opposite result. We implemented both summation rules and compared the goodness-of-fit of the resulting models. In the context of our data, the model with the summation-first rule fits better than the model with the power-first rule. For averaged data, the RMSE for the latter model was 22% larger than the RMSE of the former model. We thus adopted the summation-first rule here.

## Conclusions

Using single and dual pattern-masking paradigms, we showed that plaid pattern detection is mediated by a combination of two contrast gain control mechanisms with receptive fields whose orientations are orthogonal to each other. We also found no evidence of involvement by a mechanism with a linear plaid or a nonoriented filter in the early stages of pattern processing, and no evidence of involvement by a mechanism with late suppression.

## Supporting Information

S1 Appendix A. Implementing a Two-mechanism model for plaid detection and discrimination. B. Implementing a three-mechanism model for plaid detection and discrimination.
(DOCX)

S1 Table Individual data set obtained for all conditions. (XLSX)
