# Supplementary material for: Contrast Gain Control in Plaid Pattern Detection
Source: PLoS One. 2016 Oct 20;11(10):e0164171. doi: 10.1371/journal.pone.0164171 (PMC5072603; doi:10.1371/journal.pone.0164171)
Supplement: S1 Appendix — A. Implementing a Two-mechanism model for plaid detection and discrimination. B. Implementing a three-mechanism model for plaid detection and discrimination. (DOCX) [file pone.0164171.s001.docx]

Appendix A. *Implementing a Two-mechanism model for plaid detection and discrimination*

We fitted the data quantitatively to investigate whether a two-mechanism model is necessary and sufficient to explain the underlying mechanism for plaid detection. To simplify the model, we assumed the visual system to have matched filters for the stimuli. In this two-mechanism model, the detection of a plaid was determined by two orthogonal spiral filters with contrast gain control. Thus there were 6 *Se*-related parameters (*Se_S,S_*, *SeS_S,cS_*, *Se_S,PL_* ,*Se_cS,cS_*, *Se_cS,S_*, and *Se_cS,PL_*) and 6 *Si*-related parameters (*Si_S-S_*, *Si_S-cS_*, *Si_S-PL_* ,*Si_cS-cS_*, *Si_cS-S_*, and *Si_cS-PL_* ) for the two-mechanism model. *S* denotes spiral; *cS* denotes a counter spiral, and *PL* denotes plaid. For example, *Se_S,PL_* denotes the excitatory sensitivity from a plaid pattern in the spiral-sensitive mechanism. To better constrain the model, we set *Se_S,S_*=*Se_cS,cS_*=100, and *Si_j,k_* = *Si_k,j_*, for all *j* and *k*. This meant that the inhibition strength was symmetrical between the two mechanisms. We assumed the excitation of each filter to be narrowly tuned; thus *Se_j,k_* = 0. The S and cS components of a plaid pattern were detected by the S and cS filters, respectively. We also assumed *p,* *q’,* *q*, and *z* to be the same for these two mechanisms and fixed *q’* to one*.* Thus, with these constraints, there were 5 free parameters (*Si_S,S_*=*Si_cS,cS_*, *Si_S,cS_*=*Si_cS,S_*, *p*, *q*, and *z*) in this model.

The model parameters were optimized with a least-square method that minimized the sum of the squared difference between the data and the model predictions in dB units. Table A1 shows the root mean squared error (RMSE) for each participant. The best fits of this model are shown as smooth curves in Fig 2. The fitted parameters are shown in Table A2. To examine the stability of each parameter, we estimated a 95% confidence interval for each parameter following a procedure proposed by Bevington & Robinson (1992). We increased or decreased the value of one parameter at a time from its best value and then optimized the rest of the free parameters. This process was repeated until the change in the parameter in question increased the *X^2^* measurement for goodness-of-fit by 3.84; at a degree-of-freedom = 1, the value corresponded to 95% cumulative probability. In summary, the two-mechanism model describes our results well and the fitted parameters were also reliable.

Table A1. Two-mechanism model

|  | PCH | YSH | NP3 | NP4 | NP5 | NP6 | NP7 | AVG |
| --- | --- | --- | --- | --- | --- | --- | --- | --- |
| mean standard error from data | 1.83 | 2.07 | 2.44 | 2.35 | 2.51 | 2.78 | 2.06 | 0.58 |
| RMSE from fits | 2.16 | 2.04 | 1.96 | 2.75 | 3.00 | 2.37 | 1.69 | 1.51 |

Table A2. Fitted parameters for the best model and its 95% confidence interval for the averaged results.

|  | **Se_S-S_* ,*Se_cS-cS_* | *Si_S-S_* ,*Si_cS-cS_* | *Si_s –cS_* , *Si_cS –S_* | *p* | *q* | z |
| --- | --- | --- | --- | --- | --- | --- |
| Parameters | 100 | 62.32 | 5.59 | 3.28 | 2.97 | 1.53 |
| -95% CI | - | 0.34 | 0.09 | 0.005 | 0.004 | 0.11 |
| + 95% CI | - | 0.90 | 0.09 | 0.001 | 0.006 | 0.12 |
| SSE | 127.24 | RMSE | 1.51 | Data points | | 56 |

*Appendix B. Implementing a three-mechanism model for plaid detection and discrimination*

We tested whether a plaid-specific mechanism was necessary to explain our result quantitatively by adding a plaid mechanism that contained a matched linear filter for the plaid stimuli used in our experiment and operated in parallel with the other two oriented mechanisms. There were 21 possible parameters (*Se_S,S_*, *Se_cS,cS_*, *Se_PL,PL_*, *Se_S,cS_*, *Se_S,PL_*,*Se_cS,S_*, *Se_cS,PL_*, *Se_PL,S_*, Se_PL,cS_ , *Si_S,S_*, *Si_cS,cS_*, *Si_PL,PL_*, *Si_S,cS_*, *Si_S,PL_*, *Si_cS,S_*, *Si_cS,PL_*, *Si_PL,S_*, *Si_PL,cS_*, *p*, *q*, and *z*) in the three-mechanism model. We fixed *Se_S,S_* _,_ *Se_cS,cS_* and *Se_PL,PL_* to the corresponding image components at 100; we set *Si_j,k_* _=_ *Si_k,j_*, for all *j* and *k* as our two-mechanism model and assumed *p,* *q’* *,* *q*, and *z* to be the same for all three mechanisms and fixed *q’* to one. Furthermore, the excitatory sensitivity of a plaid filter to a spiral pattern and counter spiral pattern was fixed at 0; the excitatory sensitivity of a spiral and counter spiral filter was sensitive to the plaid components (*Se_S,PL_*=*Se_cS,PL_*=50). Thus, with these constraints, there were 7 free parameters (*Se_S,S_*=*Se_cS,cS_*=*Se_PL,PL_*=100, *Si_S,S_*=*Si_cS,cS_*, *Si_PL,PL_*, *Si_S,cS_*=*Si_cS,S_*, *Si_S,PL_*=*Si_PL,S_*=*Si_cS,PL_*=*Si_PL,cS_*, *p*, *q*, and *z*) in the three-mechanism model. A plaid pattern produced some excitation in all spiral, counter spiral, and plaid detectors, but in different amounts. Despite having two more free parameters, the SSE of the three-mechanism model showed no significant improvement over the two-mechanism model. That is, adding an extra plaid mechanism did not improve the fit of the model.

It is possible that a third mechanism characterized by something other than plaid sensitivity may be necessary. We tested this hypothesis by setting as a free parameter. Despite three extra free parameters, the SSE of the three-mechanism model showed little improvement over the two-mechanism model. The F-test for model comparison showed that the difference in SSE was insignificant for the averaged data (F (3,48)=0.94 , p=0.48) and all but one participant (YSH, F(3,48)=8.40, p<0.01). Thus, adding a third mechanism, even an arbitrary one, did not improve the goodness-of-fit in general. We thus concluded that the third mechanism was not necessary to explain our data.
